# A Previously Undiagnosed Case of Alkaptonuria in an 80-Year-Old Patient: A Case Report

**DOI:** 10.7759/cureus.35792

**Published:** 2023-03-05

**Authors:** Mohit K Singh, Faisal A Memon, Smita A Deokar, Yash Achhapalia, Ganesh R Yeotiwad

**Affiliations:** 1 Biochemistry, Hinduhrudaysamrat Balasaheb Thackarey (HBT) Medical College and Dr. R. N. Cooper Hospital, Mumbai, IND; 2 Internal Medicine, Hinduhrudaysamrat Balasaheb Thackarey (HBT) Medical College and Dr. R. N. Cooper Hospital, Mumbai, IND; 3 Surgery, Liverpool University Hospital NHS Foundation Trust, Liverpool, GBR; 4 Orthopaedics, Hinduhrudaysamrat Balasaheb Thackarey (HBT) Medical College and Dr. R. N. Cooper Hospital, Mumbai, IND

**Keywords:** ascorbic acid, nitisinone, biochemical tests, ochronosis, homogentisic acid, genetics, alkaptonuria

## Abstract

Alkaptonuria is a rare genetic metabolic disorder of autosomal recessive inheritance characterised by the accumulation of homogentisic acid in the body. It is diagnosed upon identification of characteristic symptoms, using various biochemical investigations, radiographic pictures, and a variety of specialised tests. Here we are discussing the case of an 80-year-old female patient with incidental findings of alkaptonuria. It is crucial to understand the fundamental diagnostic investigations that can be used in low-income nations or facilities where investigations like genetic testing, gas chromatography, and mass spectrometry are not readily available for the diagnosis of alkaptonuria.

## Introduction

Alkaptonuria (AKU) is a rare disease of autosomal recessive inheritance. It is generally seen in around 1 in 250,000 to 1 in 500,000 live births worldwide, but higher incidences of 20 to 25 in 500,000 live births are detected in Slovakia and the Dominican Republic [1,2]. Very few scattered cases are reported in India [3]. But according to recent case reports, there are localised higher incidences in some parts of south India [1,3-5]. Alkaptonuria is caused by a mutation in the homogentisate 1,2-dioxygenase (HGD) gene, which in turn causes the absence of homogentisate 1,2-dioxygenase, the enzyme produced mainly by hepatocytes in the liver and within the kidney. It is responsible for the conversion and breakdown of homogentisic acid (HGA), which is an intermediate in the tyrosine and phenylalanine degradation pathways and results in the accumulation of HGA. Classically, due to the excess HGA, patients pass dark urine, which, upon standing, turns brown or black. This is a feature that can be present from birth but often goes unnoticed or is ignored by the patient or their parents. This can be due to a lack of public health awareness about this condition among Indians. As this condition is so rare, it is often overlooked by clinicians as a diagnosis. Late-age diagnoses are rare. Later on in life, patients develop other symptoms, mainly due to the deposition of HGA in collagenous tissues, which is called ochronosis and ochronotic osteoarthropathy. Valvular calcifications, dark pigmentation of the skin, and sclera are also seen. Here we present a rare case report of an 80-year-old Indian female patient with incidental findings of ochronosis and scleral pigmentation. The major aim of this case report is to shine a light on this rare disease in the Indian subcontinent and how it might present in an individual in later years. We also want to discuss the availability of detection methods that can be used in any tertiary centre at a fraction of the cost when compared to the gold standard investigations. Overall, it may help in early diagnosis and finding out the actual incidence of AKU in Indian settings.

## Case presentation

**Figure 1:**
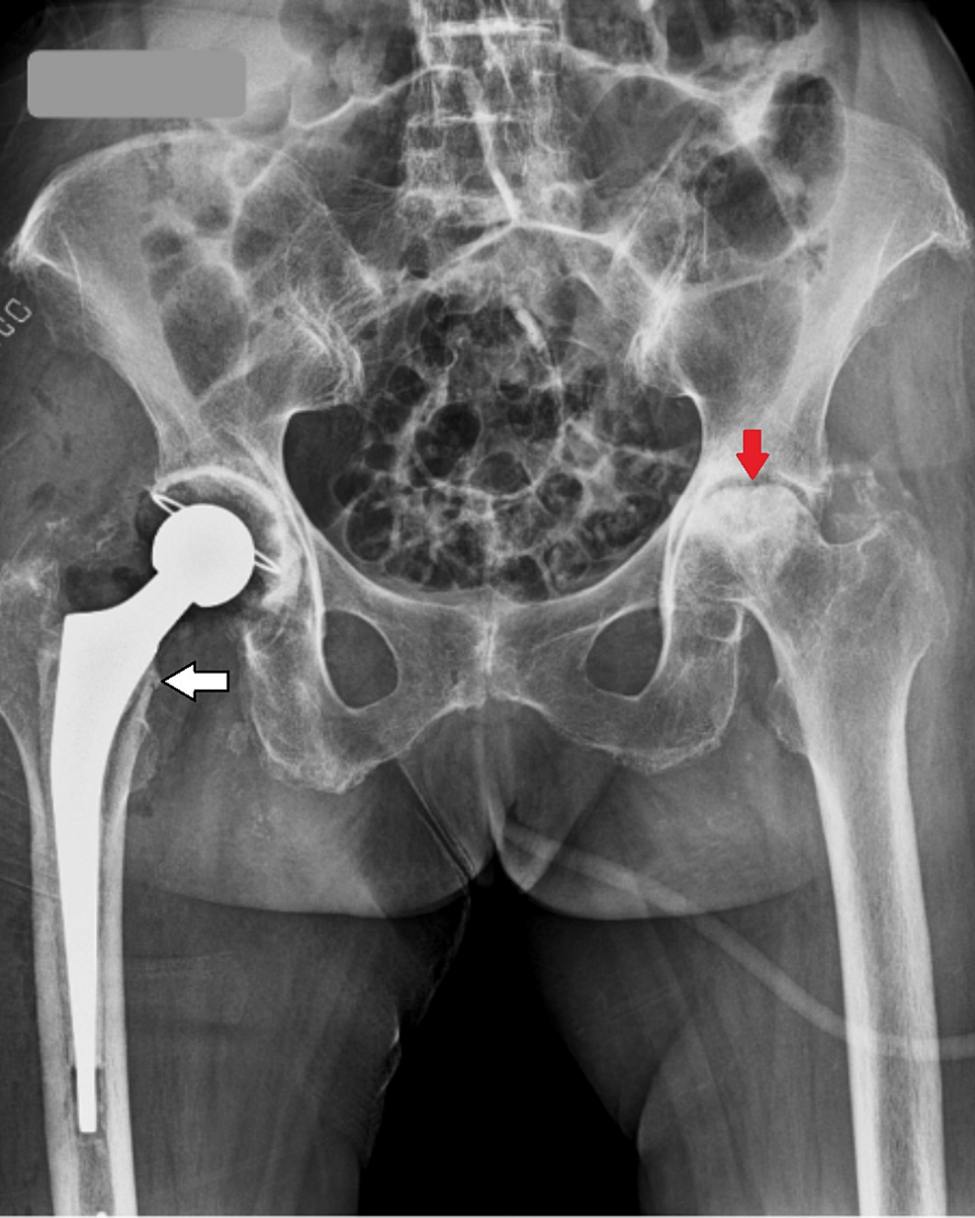
Post hemiarthroplasty X-ray of right hip joint (white arrow) with arthritic changes in left hip joint (red arrow)

**Figure 2:**
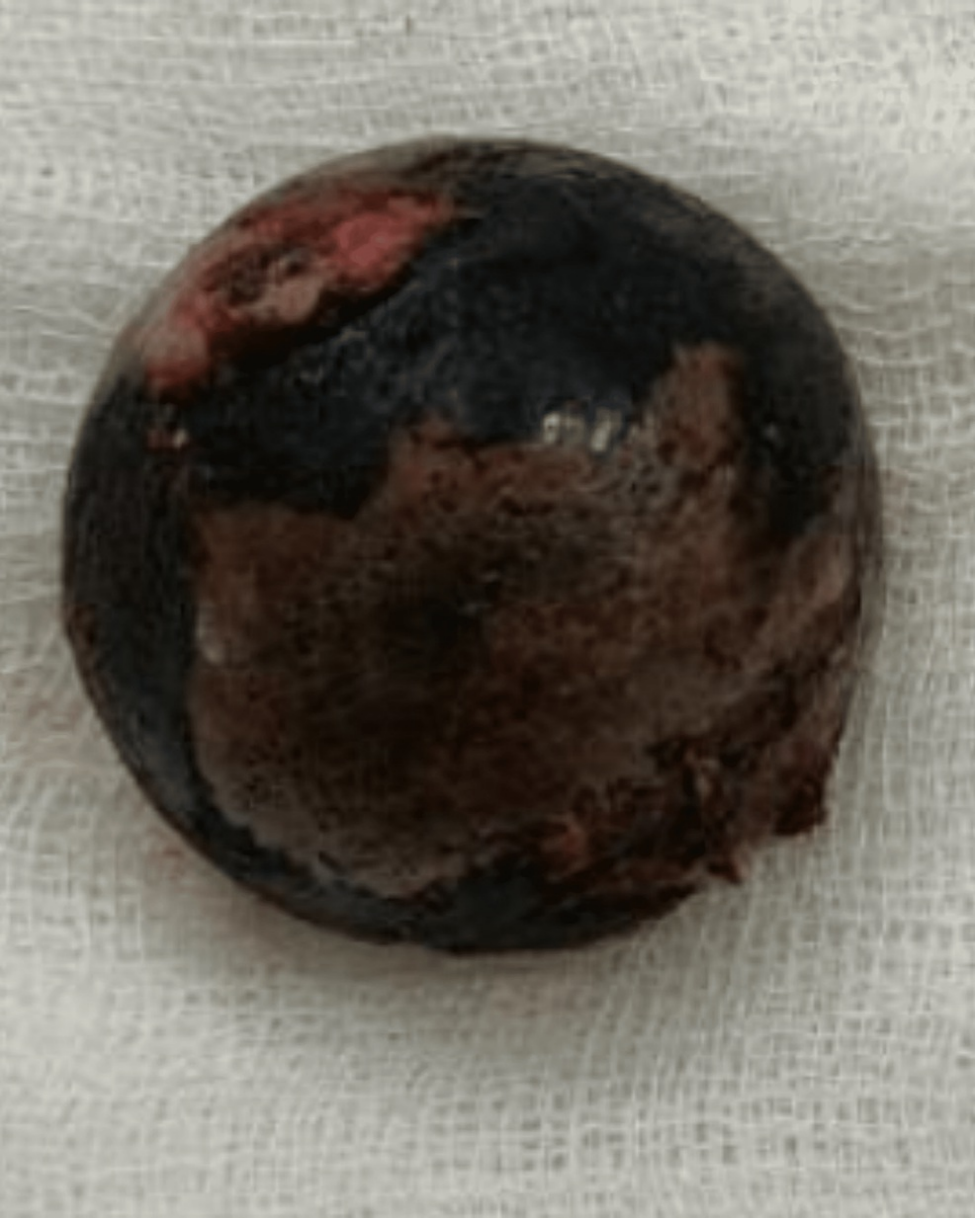
Excised black-coloured femur head

**Figure 3:**
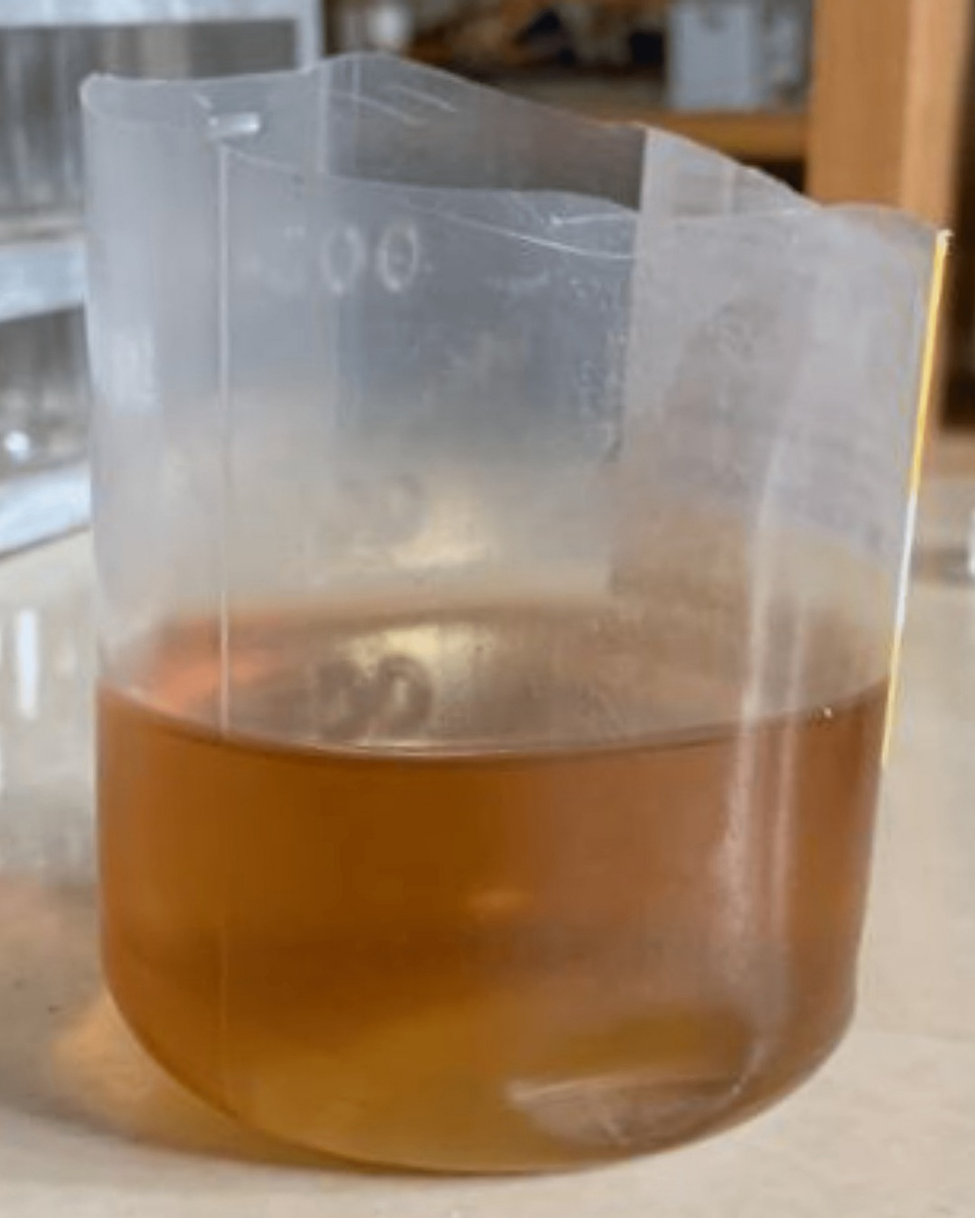
Urine darkens in colour on standing

**Figure 4:**
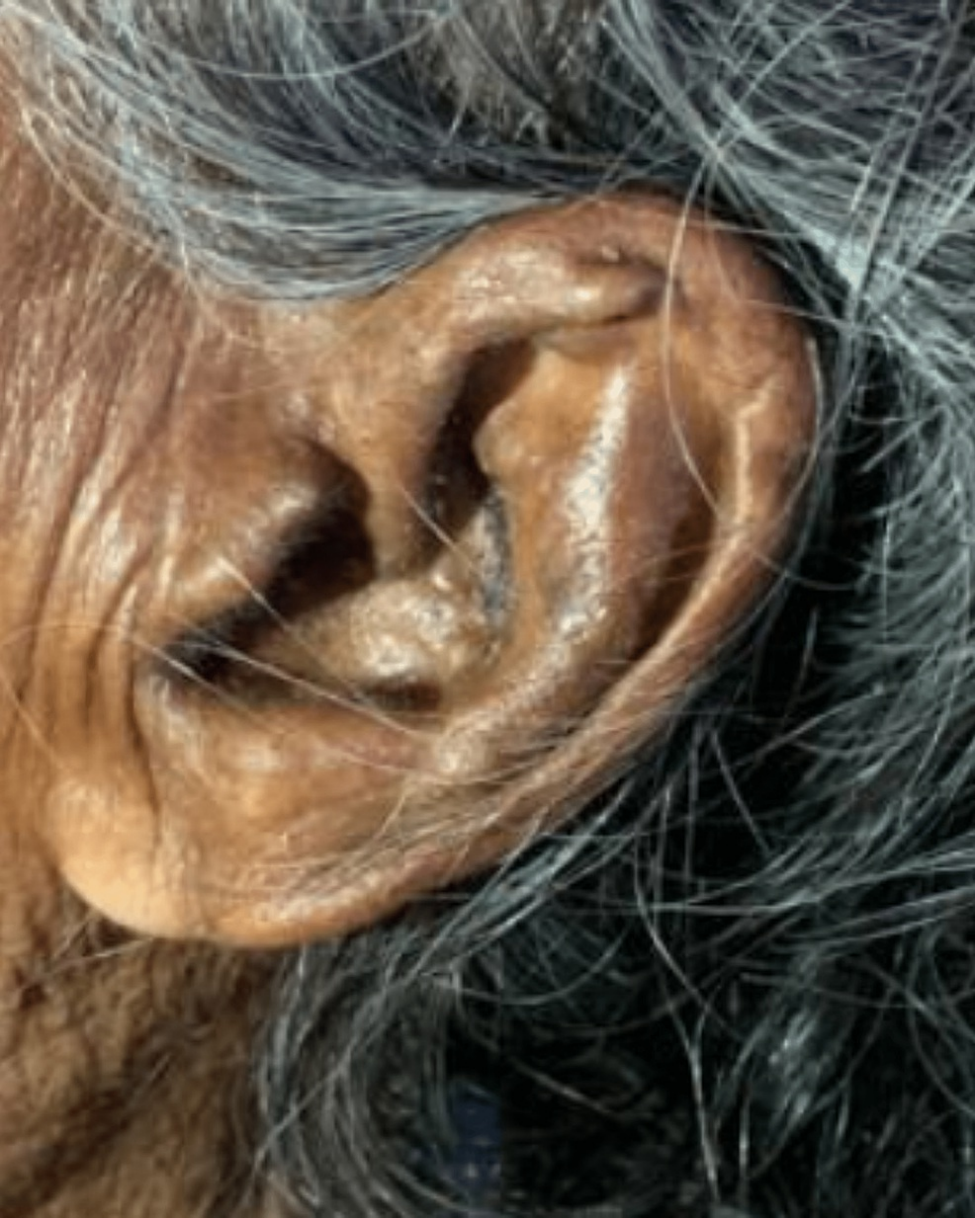
Hypertrophied ear cartilage

**Figure 5:**
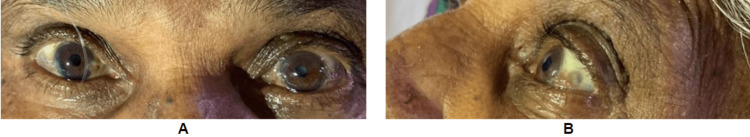
Scleral pigmentation

**Figure 6:**
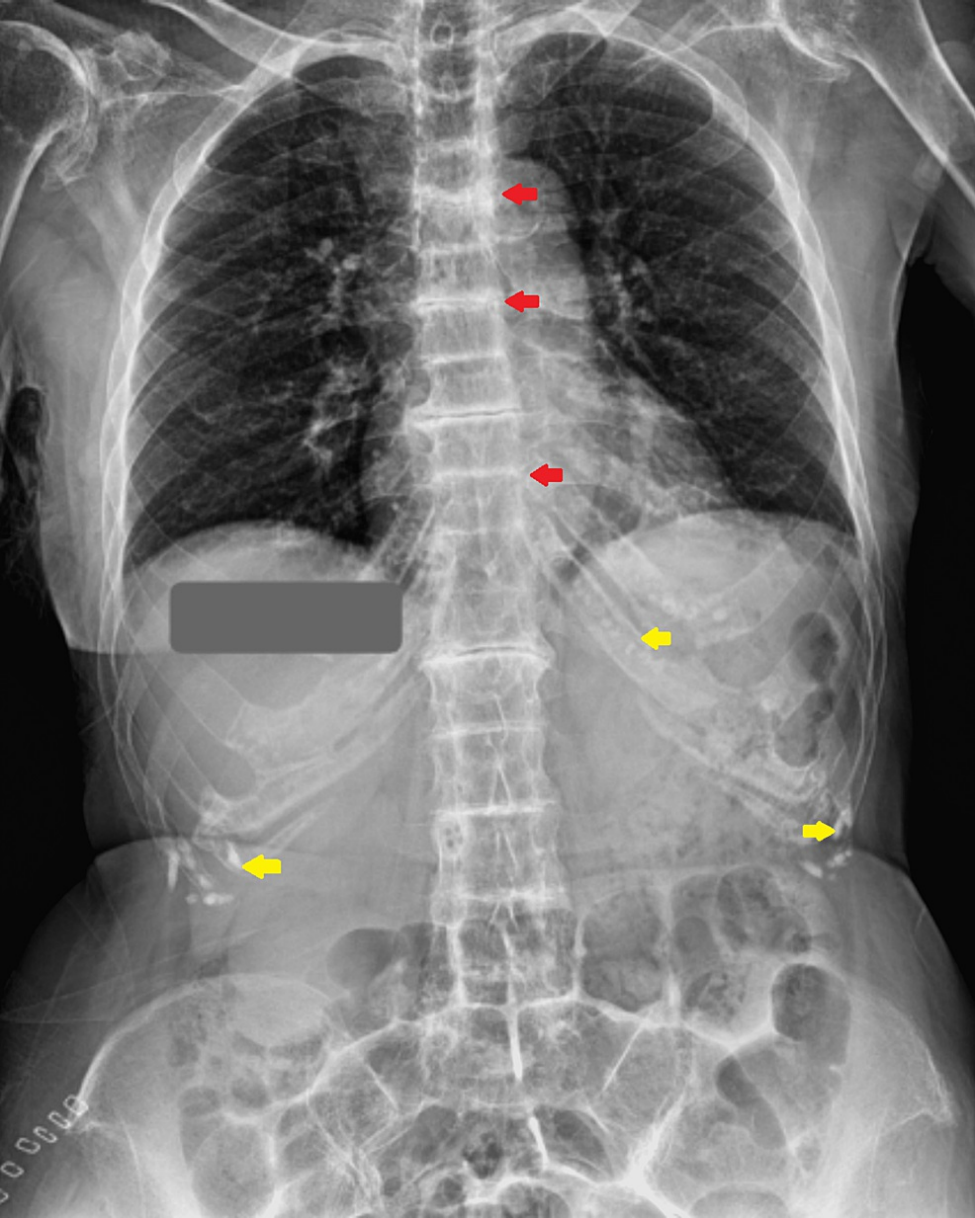
X-ray dorso-lumbar spine shows Intervertebral discs (red arrow) and costochondral calcification (yellow arrow) with bamboo spine appearance

**Figure 7:**
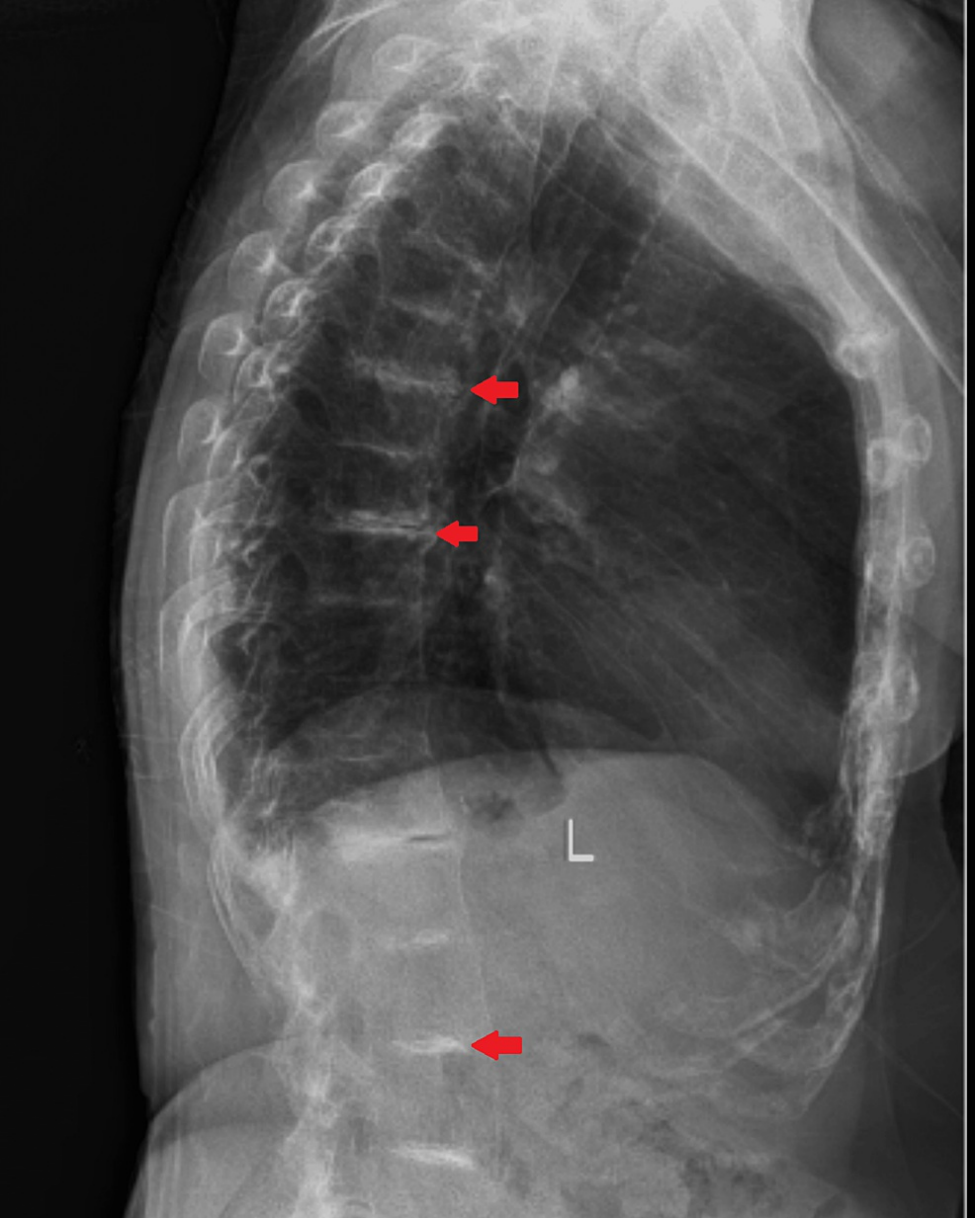
X-ray dorso-lumbar spine shows intervertebral discs with calcification (red arrows), lateral view

An 80-year-old female patient came to the hospital with complaints of right leg pain and inability to bear weight, which had an associated history of falling a few days ago. She gave a history of hypertension, well controlled by medication. Local examination and the X-ray report revealed a fracture of the right neck of the femur without any nerve or vessel injury. Patient underwent right total hip replacement a few days later (Figure 1). Intraoperatively, it was discovered that the joint capsule, joint surfaces, ligaments, and tendons had black pigmentation. The excised femur head was also black in colour (Figure 2). Postoperatively, it was noted that the urine collected in the urine bag was turning black on standing, and a diagnosis of alkaptonuria was therefore suspected (Figure 3). A thorough re-examination of the patient revealed hypertrophy of the ear cartilage and black pigmentation (Figure 4). On ocular examination, scleral pigmentation was also noted (Figure 5). On an X-ray of the chest, calcification of the costochondral junction was seen. On X-rays of the spine, calcification of the intervertebral disc was also seen (Figures 6, 7).

**Figure 8:**
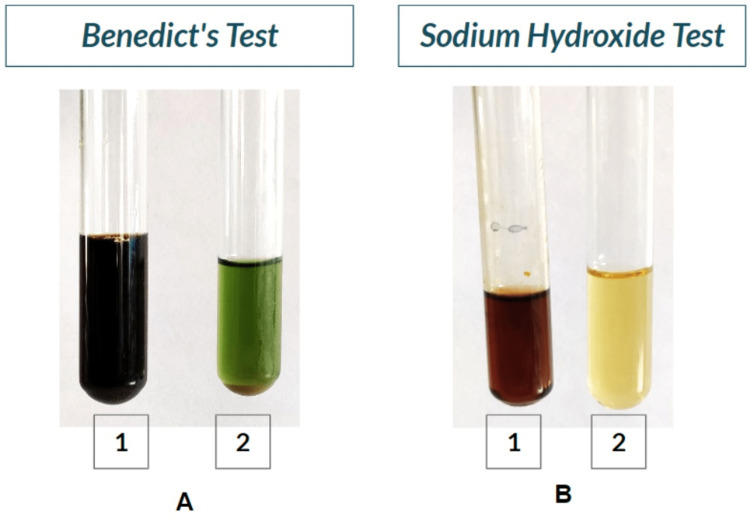
Positive Benedict's test and sodium hydroxide test. A: Benedict's test- test tube 1 shows positive test on patient's urine sample and test tube 2 shows negative test in control tube. B: Sodium hydroxide test- test tube 1 shows positive test on patient's urine sample and test tube 2 shows negative test in control tube.

**Figure 9:**
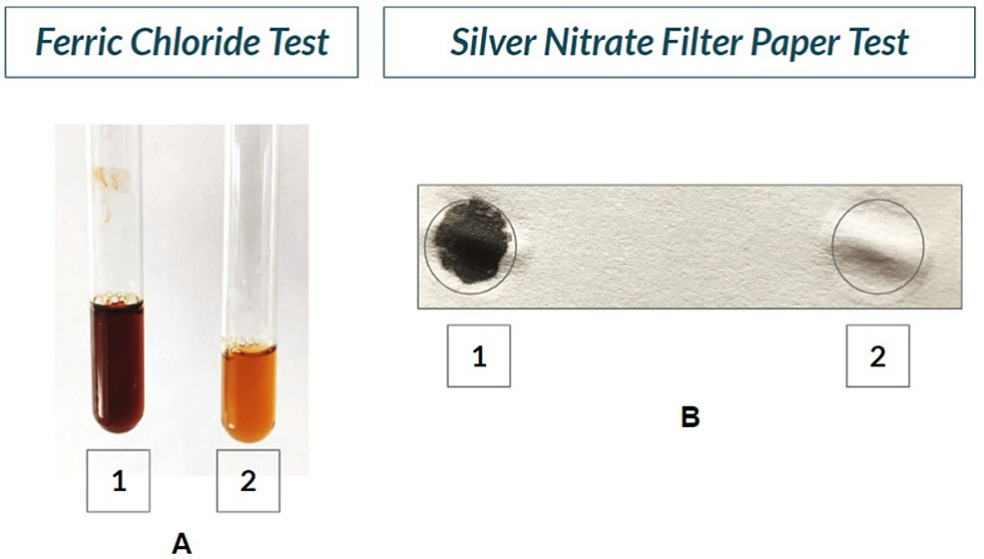
Positive ferric chloride test and silver nitrate filter paper test. A: Ferric chloride test- test tube 1 shows positive test on patient's urine sample and test tube 2 shows negative test in control tube. B: Silver nitrate filter paper test- circle 1 shows positive test on patient's urine sample and circle 2 shows negative test on control urine sample.

**Figure 10:**
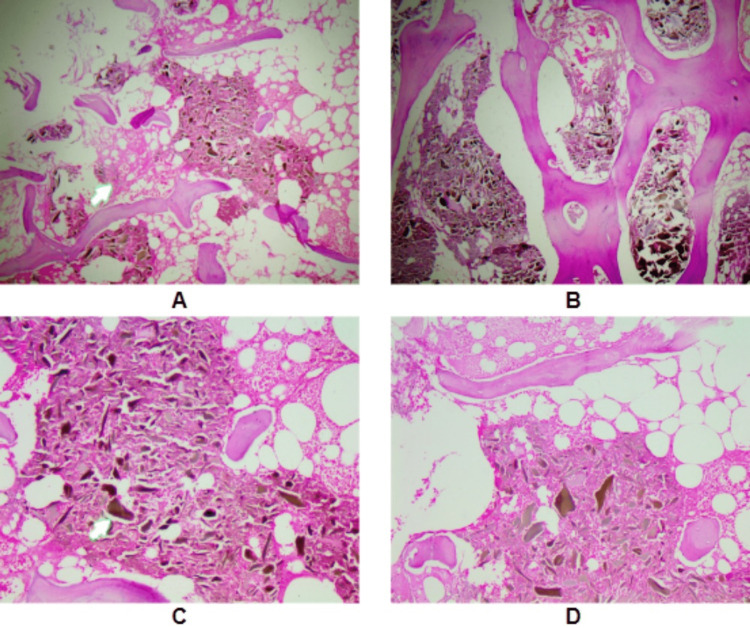
Ochronosis seen on histopathological examination of excised femur head with hematoxylin and eosin staining. A: 10X magnification B, C, D: 40X magnification

Urine was collected and sent for biochemical investigations, as the patient couldn't afford gas chromatography, mass spectrometry or genetic testing. Table 1 shows the physical characteristics of the collected urine sample. Table 2 shows the biochemical investigations done on the sample (Figures 8, 9). Ochronosis is also seen on the histopathological examination of the excised femur head (Figure 10). 

**Table 1:** Physical characteristics of the urine sample

Characteristic	Observation
Colour	Dark brown
Appearance	Slightly Turbid
Odour	Ammoniacal
pH	Acidic
Specific Gravity	1.015

**Table 2:** Biochemical tests performed on the urine sample Tests 1 to 4 were confirmed on urine dipstick

Test	Results
Sulphosalicylic acid Test	Negative for Protein
Rothera's Test	Negative for Ketone Bodies
Fouchet's Test	Negative for Bile Pigments
Hays Sulfur Test	Negative for Bile Salts
Benedict's Test	Strongly positive with Black supernatant and yellow precipitate. High chances of Homogentisic Acid presence.
Ferric Chloride Test for Homogentisic Acid	Transient blue colour obtained which rapidly turned to brown. Homogentisic Acid present.
1M NaOH Test for Homogentisic Acid	Brown colour obtained. Homogentisic Acid present.
5% Silver Nitrate soaked ﬁlter paper Test	Black pigmentation. Homogentisic Acid present.

All the results pointed towards a diagnosis of alkaptonuria. When asked about their family history, the patient had no more information regarding relatives who had experienced similar symptoms or concerns. It is very unusual to remain undiagnosed this late in life with all these presenting signs and symptoms. The patient was then started on 1.5 grams of vitamin C daily for the next month.

## Discussion

Alkaptonuria, an autosomal recessive disorder, is a rare inborn error of metabolism that affects around 1 in 250,000 to 1 in 500,000 live births worldwide and results in HGA oxidase enzyme deﬁciency in the liver and kidneys. It usually presents in later stages with a wide variety of systemic involvement [6]. The enzyme deficiency leads to accumulation of HGA, which forms a part of the metabolic pathway of phenylalanine and tyrosine, leading to the formation of a melanin-like pigment that has a high afﬁnity for cartilage and other connective tissues, forming a brown-ocher colour. Hence this disease is also called endogenous ochronosis [7]. It can manifest in multiple ways. In children, it commonly presents as the darkening of urine after a period of rest or exposure to an alkaline environment, often seen as black spots in diapers (more commonly seen in males due to prostate involvement). In adults, usually by the fourth decade of life, it can affect numerous systems (Table 3) [8].

**Table 3:** Various systemic manifestations of alkaptonuria.

System	Signs and Symptoms
Aural	Blackish discolouration of earwax, bluish coloration of outer ear cartilage.
Dermatological	Brownish discolouration of axillary and malar regions, papules and blackened blue vesicles in the palmar and plantar regions.
Cardiovascular	Pigmentation and calcification of aortic and mitral valves, endocardium, aorta and coronary arteries.
Genitourinary	Alteration of urine color, renal, prostatic lithiasis.
Ocular	Pigmentation of sclera (Osler's sign), cornea, conjunctiva, eyelids.
Skeletal	Ochronosis arthropathies common in hip, knee, shoulders; narrowing and calcification of lumbar intervertebral spaces; rupture of ligaments and tendons.
Others	Gallbladder and salivary gland stones, dysphagia

It is usually diagnosed by clinical history, but a urine test for HGA is the gold-standard test to diagnose alkaptonuria. The amount of homogentisic acid in the 24-hour urine is detected via gas chromatography-mass spectrometry analysis [9,10]. Genetic analysis of the deficient enzyme is considered the standard investigation to help in family counselling [9]. It is imperative to know about biochemical investigations, which can be done in low-income countries where genetic testing or the “gold standard” testing of HGA detection in urine via gas chromatography or mass spectrometry is not readily available for the diagnosis of alkaptonuria. 

Trivedi (2020) [1] has discussed various comprehensive technical details of biochemical methods with reagent preparations so that any primary health centres or small labs will be able to detect positive samples with confidence and contribute to the diagnosis of AKU. These biochemical tests are performed on fresh urine samples [1,11]. HGA gives a variety of positive colour reactions. The phenols of HGA form a bluish complex with Fe3+ in ferric chloride solution, and hence urine containing HGA yields a transient blue colour. HGA in an alkaline medium, like NaOH, undergoes oxidative polymerization to quinone derivatives, which are black to brown in colour. HGA also reduces alkaline ammoniacal silver nitrate solution to metallic silver, which is black-coloured. In Benedict's test, reducing sugar and the non-sugar-reducing substance in an alkaline medium reduces copper sulphate to cuprous oxide. Colour depends on the quantity of the reducing substance. Hence, it gives black supernatant with yellow precipitate in addition to HGA. 

Although this condition does not reduce life expectancy, it significantly affects quality of life. So far, there is no curative pharmacological treatment for alkaptonuria. For arthropathies, non-steroidal anti-inflammatory drugs (NSAIDs) and physiotherapy have proved to be of signiﬁcant beneﬁt; however, surgery may be required in some cases as well. Dietary restrictions of proteins, especially tyrosine and phenylalanine amino acids (soy products, milk and dairy products, eggs, almonds, peanuts, etc.). The formation of the melanin-like HGA polymer is an oxidative process via benzoquinone acetic acid (BQA). Ascorbic acid is an antioxidant that reduces BQA production and influences the development of polymers. A high dose of vitamin C, 500 mg two to three times daily, can be given for its antioxidant purposes, which prevents the oxidation of HGA, although its efficacy is highly debatable and needs to be evaluated further [12-14].

Several studies have investigated the use of nitisinone in the treatment of AKU. Some of the most significant findings are from a study conducted by the National Alkaptonuria Centre in the United Kingdom, which showed that nitisinone treatment led to a significant reduction in HGA levels and a slowing of disease progression in AKU patients [15]. Other studies have also demonstrated the efficacy of nitisinone in reducing HGA levels and improving clinical outcomes in AKU patients [16-19]. Nitisinone is a small molecule inhibitor of 4-hydroxyphenylpyruvate dioxygenase (HPPD), an enzyme that converts 4-hydroxyphenylpyruvic acid to HGA in the tyrosine catabolic pathway. By inhibiting HPPD, nitisinone reduces the amount of HGA that is produced, leading to a decrease in its accumulation in the body. In addition to its use in AKU, nitisinone is also used in the treatment of hereditary tyrosinemia type 1 (HT-1), a rare genetic disorder that affects the liver. Overall, nitisinone has shown great promise as a treatment for AKU and other conditions. Ongoing research will continue to explore the mechanisms of action of nitisinone and its potential use in other diseases, with the ultimate goal of improving patient outcomes and quality of life [20].

In the Indian subcontinent, the actual incidence of AKU is still unknown, which could be mainly due to the lack of a central database and lack of awareness among patients and clinicians about this condition. Trivedi and Haridas (2015) [5] have discussed five cases of AKU from a single family, spanning two generations, in Karnataka, India. The cases were in the age range of two and a half years old to 48 years old. In the case report, only two patients showed signs and symptoms of the disease, manifesting as arthritis and black pigmentation in one and psychological problems in the other, while the rest three had only positive urine tests for AKU. Tharini et al. (2011) [3] also discussed a case of a 51-year-old male from Chennai, India, with a history of darkening urine, black pigmentation of the sclera, and hip and knee involvement.

It is essential to have a multidisciplinary approach in patients with AKU, with regular monitoring for bone, ear, eye, and heart health.

## Conclusions

It is critical to understand the biochemical investigations that can be performed in primary health care centres or small labs in low-income countries where genetic testing, gas chromatography, or mass spectrometry are not readily available for the diagnosis of alkaptonuria due to cost to the patient or a lack of infrastructure. Early detection and treatment of such entities are critical in reducing difficulties later in life, and management is attainable.
